# Improving Malaria Case Management and Referral Relationships at the Primary Care Level in Ghana: Evaluation of a Quality Assurance Internship

**DOI:** 10.9745/GHSP-D-23-00050

**Published:** 2023-12-22

**Authors:** Amos Asiedu, Rachel A. Haws, Akosua Gyasi, Paul Boateng, Keziah Malm, Raphael Ntumy, Lolade Oseni, Gladys Tetteh

**Affiliations:** a U.S. President's Malaria Initiative, Impact Malaria Project, Accra, Ghana.; b Department of International Health, Johns Hopkins Bloomberg School of Public Health, Baltimore, MD, USA.; c National Malaria Control Programme, Accra, Ghana.; d U.S. President's Malaria Initiative, Impact Malaria Project, Jhpiego, Baltimore, MD, USA.

## Abstract

In Ghana, Community-based Health Planning and Services (CHPS) compounds managed by trained nurses and midwives called community health officers (CHOs) play a major role in malaria service delivery. With heavy administrative burdens and minimal training in providing patient care, particularly for febrile illnesses, including malaria, CHOs struggle to comply with the World Health Organization's test, treat, and track initiative guidelines and appropriate referral practices. A clinical training and mentorship program was implemented for CHOs to prevent and manage uncomplicated malaria and offer appropriate pre-referral treatment and referrals to district hospitals. Medical officers, pharmacists, midwives, health information officers, and medical laboratory scientists at 52 district referral hospitals were trained as mentors; CHOs from 520 poorly performing CHPS compounds underwent a 5-day internship at their assigned district referral hospital to improve knowledge and clinical skills for malaria case management. Three months later, mentors conducted post-training mentoring visits to assess knowledge and skill retention and provide ongoing on-the-job guidance. Significant percentage-point increases were observed immediately post-internship for history taking (+12.0, 95% confidence interval [CI]=8.3, 15.1; *P*<.001); fever assessment (+24.9, 95% CI=20.9, 29.3; *P*<.001); severe malaria assessment and referral (+32.0, 95% CI=28.2, 35.8; *P*<.001); and knowledge assessment (+15.8, 95% CI=10.0, 21.3; *P*<.001). Three months later, a third assessment revealed these gains were largely maintained. Analysis of national health management information system data showed statistically significant improvements in testing, treatment, and referral indicators at intervention CHPS compounds after the intervention that were not observed in comparison CHPS compounds. This training and mentorship approach offers a replicable model to build primary care provider competencies in malaria prevention and management and demonstrates how developing relationships between primary care and first-level referral facilities benefits both providers and clients. More methodologically rigorous studies are needed to measure the impact of this approach.

## Abstract (precis)

The authors report that an innovative internship and mentoring program for community health officers in Ghana was associated with improved knowledge and skills related to malaria management, including timely, appropriate referrals for severe cases.

## Teaser

**Key Findings:**

- Community health officers (CHOs) who participated in the internship program increased their knowledge and skills in malaria case management and diagnosis and treatment of febrile illnesses.
- Three months after the internship, interns retained clinical competency gains in history taking, severe malaria assessment and referral, and fever assessment, but competency in managing acute respiratory infections declined slightly.
- A novel feature of the CHO internship was facilitating supportive relationships between CHOs and mentors based at each CHO's assigned district referral hospital, which promoted strong relationships that are essential to timely referral and effective management of severe malaria.

**Key Implications:**

- If these promising findings are confirmed by an impact study, malaria program managers might consider expanding training for community health nurses so they are equipped with the requisite clinical skills to diagnose and manage febrile illnesses, including malaria, as well as to make timely and appropriate referrals for severe malaria.
- Policymakers could increase the involvement of non-physician cadres in providing malaria case management services by offering appropriate clinical training and support.
- Policies to ensure that strong, supportive relationships are in place between referral facilities and primary health centers can support effective referrals.

## BACKGROUND

Ghana and other sub-Saharan African countries have made remarkable progress in reducing the burden of disease associated with malaria.^1^ In Ghana, strong government commitment and significant donor funding have fueled decreases in malaria morbidity and mortality of more than 50% between 2000 and 2015; Ghana also has one of the highest coverage rates for 3 or more doses of intermittent presumptive treatment for pregnant women (IPTp3+) among sub-Saharan African countries at 60.1% in 2019, well above the average of 35% among 35 African countries implementing IPTp.^2^^,^^3^ Since 2014, Ghana has mandated laboratory confirmation of cases via microscopy or malaria rapid diagnostic test (mRDT), which has led to remarkable improvement in laboratory confirmation of cases: 93.7% of suspected outpatient cases of malaria were tested in 2019, up from 73.5% in 2014.^4^ Still, Ghana remains among the 6 African countries that account for more than half of all malaria-related deaths, as progress in many sub-Saharan African countries has stalled since 2015, prompting the World Health Organization (WHO) to introduce the “High burden to high impact” approach to overcome stagnation and ensure continued progress.^5^^,^^6^ As in other African countries, quality gaps in the diagnosis and treatment of malaria, particularly in childhood, complicate malaria control efforts in Ghana.^7^ As a result, malaria remains a leading cause of morbidity and mortality in Ghana, particularly among children aged younger than 5 years. From 2015 to 2020, 39% of all outpatient visits and 23% of hospital admissions were attributable to malaria.^8^

Community-based Health Planning and Services (CHPS) compounds provide primary health care in Ghana, representing the first point of entry to the health care system and playing a major role in malaria case management and primary care more generally.^9^ Unique to the Ghanaian context, the CHPS concept is a strategy for achieving universal health coverage of essential primary health services at the community level. With more capabilities than a typical health post, each CHPS compound serves a well-demarcated catchment population, or zone, of approximately 5,000 people (5–8 communities within a radius of approximately 4 km). Each CHPS compound comprises accommodation for a community health officer (CHO) and a service delivery point.

Each CHPS is managed by a CHO, a trained, salaried nurse or midwife who has undergone an additional 2-week training. CHOs provide a range of services, often via outreach visits outside of the compound, including prevention and management of diarrhea, malaria (including key interventions to prevent malaria in pregnancy, including IPT), acute respiratory infections, and childhood illness, as well as health education, immunization, comprehensive family planning services, and referrals, many at the household level.^10^ Each CHPS has multiple CHOs (at least 2) but only 1 main prescriber. Community health volunteers work in the community, but not at the CHPS, to support the CHOs with community mobilization and education. At CHPS compounds, CHOs also diagnose severe malaria cases, provide pre-referral treatment for serious malaria cases (rectal artesunate for children aged younger than 5 years), and facilitate timely referral to the district hospital.

Several different nursing and midwifery cadres serve as CHOs, including enrolled nurses (clinical certificate), community health nurses (public health certificate), registered nurses (diploma), and midwives. Nursing and midwifery curricula in Ghana only teach general malaria management and do not cover current malaria guidelines. There is wide variation in nursing and midwifery pre-service training, particularly for malaria case management. Nurses and midwives receive clinical pre-service training in malaria case management only if their practical includes a qualified preceptor and receive clinical in-service training only if they are posted to a health center or hospital with a qualified preceptor. CHO training adequately prepares them for their administrative management responsibilities but only offers orientation to malaria in pregnancy interventions; CHOs have no access to in-service malaria case management training. With minimal orientation about quality diagnosis, treatment, and referral for malaria, CHOs are expected to diagnose malaria with mRDTs and treat uncomplicated cases using artemisinin-based combination therapies (ACTs). They may struggle to comply with WHO test, treat, and track guidelines, particularly for patients who present with fever but have a negative mRDT result.^11^^–^^13^

CHOs receive adequate training for their administrative management responsibilities but have no access to in-service malaria case management training.

Considerable overlap exists in definitions of different strategies to improve the performance of primary health care workers in low- and middle-income countries, and there is no agreement on which approaches are most effective, though supervision approaches are the most studied.^14^ Several reviews suggest that supervision—when a senior professional from a higher level of the health system audits and/or observes a health care worker to ensure their work is completed correctly—can improve health worker performance, motivation, and quality of care.^15^^–^^17^ Supportive supervision can be described as regular, direct personal contact to guide, support, and assist health care workers in developing competencies. Outreach training and supportive supervision for malaria is a strategy to improve malaria case management using standardized checklist-based tools alongside on-the-job training, but in Ghana, these visits are currently limited to health centers and hospitals and may not occur quarterly as recommended.^18^

Mentorship approaches have been increasingly incorporated alongside supportive supervision into quality improvement interventions in other health areas, including emergency obstetric and newborn care,^19^ HIV care,^20^^,^^21^ and clinical governance,^22^ but there are no published studies of mentorship for malaria case management. Mentorship refers to a “sustained, collaborative relationship” promoting broad skills transfer from an experienced individual to a less experienced mentee to improve performance and support professional development and growth; ideally, mentoring includes role modeling and psychosocial support.^23^ Mentors improve mentees' skills via on-site observation of case management, providing individualized feedback to the provider.

A review of 52 studies from sub-Saharan Africa found that mRDTs were generally used well, though compliance with test results was variable, especially in the formal health care sector.^24^ Targeted efforts utilizing supportive supervision, didactic approaches such as high-quality in-service training, audit with feedback, quality improvement, and technological tools like short message service are potential strategies to improve adherence to malaria case management and differential diagnosis for febrile illnesses in low-resource settings.^25^^,^^26^ A review of health worker performance found that supervision plus audit-with-feedback techniques effectively reinforced in-service training, and more complex support interventions were more effective than single interventions.^27^ Efforts to improve the quality of referrals—crucial for severe malaria—tend to focus on establishing clear referral pathways, protocols, and guidelines (including administration of rectal artesunate), supported by supply-side strategies to improve efficiency and appropriateness of referrals through logistical or technological means and demand-side client education and transport schemes.^28^^,^^29^ These efforts often overlook the contribution of poor collaboration and interpersonal communication between primary health care workers and higher-level facilities to suboptimal case management and referral practices.^29^ No peer-reviewed studies have explored how mentorship might enhance interpersonal and interfacility relationships, particularly when mentors come from higher levels of the health system.

Building on an internship model to improve the quality of malaria case management developed under the U.S. President's Malaria Initiative MalariaCare Project in 2016,^30^ the U.S. President's Malaria Initiative Impact Malaria project (Impact Malaria) developed an internship program for CHOs as a means of extending preceptor training to the primary care level via a mentorship and supportive supervision model. The internship focused on recognition and treatment of febrile illnesses and aimed to improve CHOs' knowledge and skills in key areas related to prevention and prompt management of uncomplicated malaria and prompt referral with appropriate pre-referral treatment (rectal artesunate for children aged younger than 5 years) for severe malaria. The program included support during and after the internship from district-level medical professionals. Impact Malaria then measured CHOs' knowledge and clinical skills, retention of knowledge and skills over time, whether key facility-level malaria case management indicators changed after the internship, and intern satisfaction.

An internship program for CHOs was developed to improve their knowledge and skills related to prevention and prompt management of uncomplicated malaria and referral with pre-referral treatment for severe malaria.

## CHO INTERNSHIP AND MENTORING

### Selection of CHPS Compounds and CHOs

In collaboration with the National Malaria Control Programme, Impact Malaria established a set of criteria to select CHPS compounds for the intervention, guided by the WHO High Burden to High Impact approach, which uses data strategically to focus on settings with a high burden of malaria to better target limited resources.^6^ The Impact Malaria study team conducted quantitative data analysis using routine data from January 2020 to June 2021 from Ghana's national health management information system (HMIS), including indicators such as total admissions, malaria deaths, and malaria case fatality rates. Ghana is among few countries that can correctly track these indicators because facilities keep a consulting room register in addition to the outpatient department register that tracks suspected cases (provisional diagnosis), positive and negative results (microscopy and RDT), new/old cases, differential diagnosis, pregnancy status, and prescribed antimalarial. Other countries may resort to estimation because they must combine and reconcile records from the outpatient department register and the laboratory register to calculate these same indicators, with higher likelihood of error.

**FIGURE 1:**
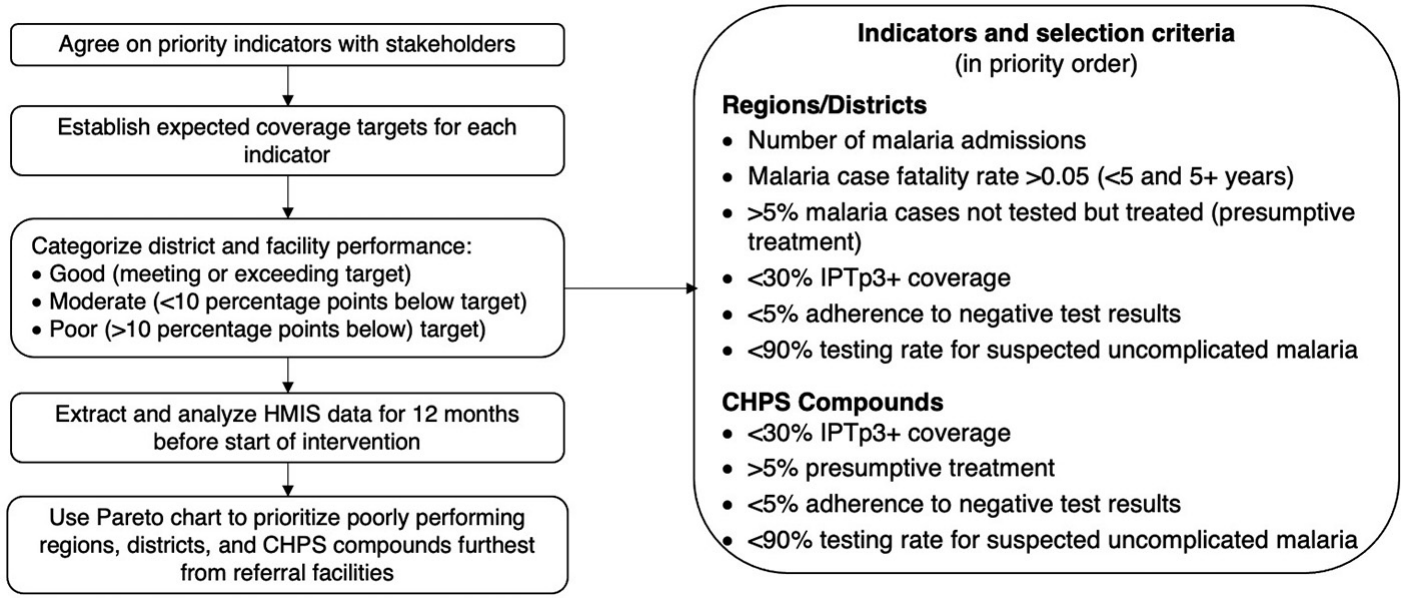
Selection of CHPS Compounds for the Impact Malaria Community Health Officer Internship and Mentoring Program in Ghana Abbreviations: CHPS, Community-based Health Planning and Services; HMIS, health management information system; IPTp3+, intermittent preventive treatment of malaria for pregnant women, 3+ doses.

Using a Pareto chart, which is a specialized bar chart prioritized by frequency, the study team identified districts with case fatality rates above the regional average and in each district prioritized CHPS compounds with lower malaria testing rates (lower than 90%), higher than 5% presumptive treatment (treatment with antimalarials without testing), lower than 5% adherence to negative test results, and lower than 30% coverage of the recommended 3 or more doses of IPTp (Figure 1).^31^ From the 10 regions targeted (all of Ghana's regions at the time), the study team selected 5 to 6 districts per region and 10 CHPS compounds per district to participate in a CHO internship program at the district referral hospital. The team selected 5 districts in most regions, but in Greater Accra and North East Ghana, which have high case fatality rates, they selected 6 districts per region.

After analyzing data from all CHPS compounds (N=2,444), the study team selected 520 CHPS compounds. Distance from each CHPS to the district referral hospital was also computed, and those CHPS compounds further from referral facilities were prioritized (this varied by region due to differences in geography and catchment area). The CHO who served as the primary prescriber at each of the 520 poorly performing CHPS compounds was selected to participate in the internship. During the regional engagement session and the first encounter with each selected CHPS, the team presented a facility performance scorecard to introduce the audit-with-feedback component of the intervention, outlining why the CHPS and its primary prescriber CHO had been selected for the internship.

### Implementation of CHO Internship and Mentoring

**FIGURE 2:**
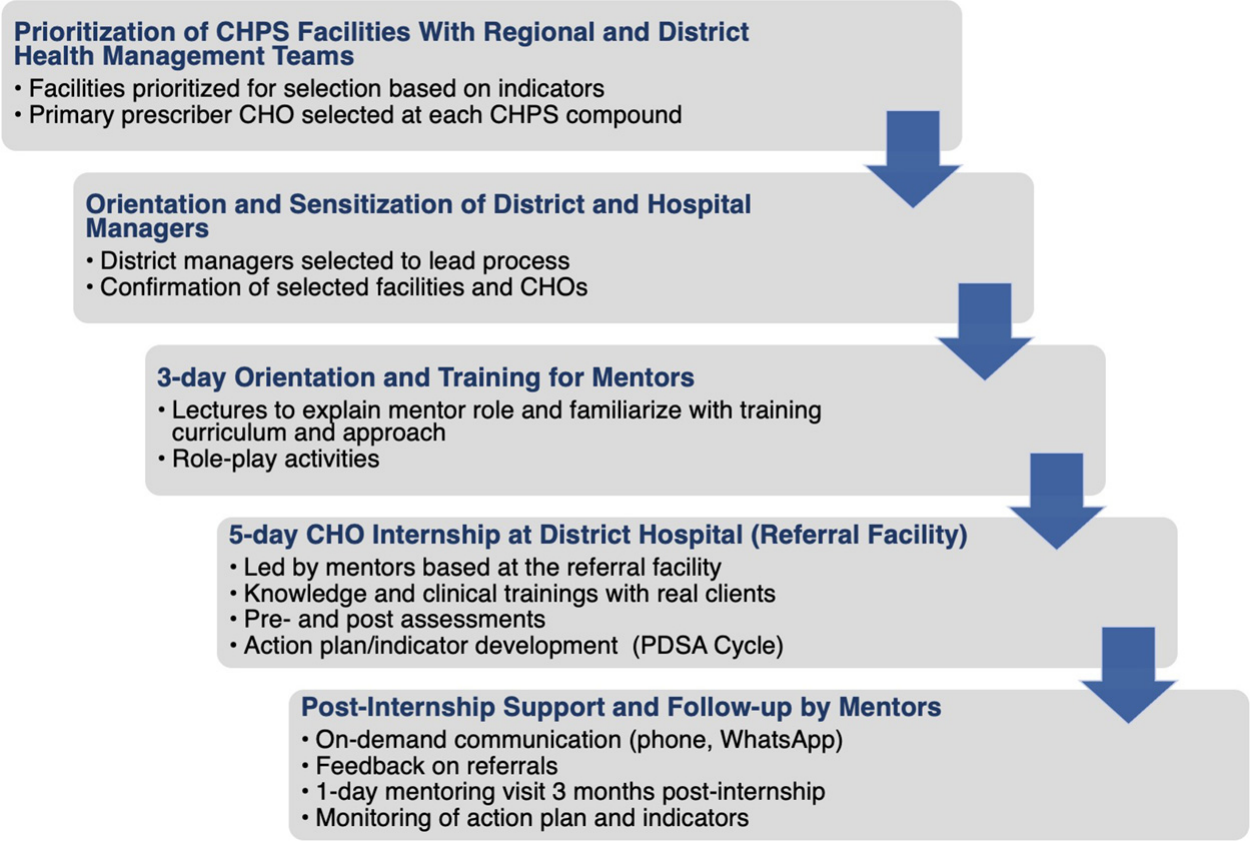
Framework and Process for Implementation of the Impact Malaria Community Health Officer Internship and Mentoring Program in Ghana Abbreviations: CHO, community health officer; CHPS, Community-based Health Planning and Services; PDSA, plan-do-study-act.

The CHO internship and mentoring program aimed to address known gaps in knowledge and skills in the management of febrile illnesses, particularly uncomplicated malaria, pre-referral management of severe malaria, and prevention and management of malaria in pregnancy. The internship also sought to forge productive mentoring relationships between interns and mentors to provide improved pre-referral treatment for severe malaria cases, prompt and appropriate referral for severe malaria cases, and ongoing mentoring and supportive supervision for CHOs. Figure 2 outlines the implementation framework for the CHO internship.

A medical officer, a midwife, and a medical laboratory scientist from each district referral hospital, as well as the health information officer and pharmacist from the district health management team, were selected as district-level mentors and oriented to the project as a mentor team. Mentors were selected based on cadre (e.g., medical doctors were selected to lead clinical knowledge and skills components) and years of experience at the referral facility or with the management team. Mentor training lasted 3 days and included lectures on content, CHO training curriculum, mentorship, mentoring techniques, and facilitation, as well as interactive role-play sessions to demonstrate how they would employ these techniques at facilities. A total of 281 district-level mentors (4–5 per district) were trained to lead implementation of the internship at the district referral hospital and provide subsequent mentoring and supportive supervision. An additional 52 district referral hospital managers (1 per district referral hospital) were trained to oversee and provide needed support for internship activities in their facility but did not engage in direct mentoring of interns. The district-level malaria focal person served as coordinator of the internship in each district.

The 520 selected CHOs then attended a 5-day internship at their assigned district referral hospital, where the trained district-level mentors provided mentoring and supportive supervision. A total of 52 internship sessions were held over 10 working days in August 2021. The district health management team, in collaboration with Impact Malaria, assigned interns in groups of 10 to rotate daily through different units (e.g., outpatient department, laboratory, antenatal clinic, and health information) of the district hospital. The 3-hour morning sessions focused on knowledge-based training, and the 5-hour afternoon sessions focused on clinical practice in small groups with real clients on skills including client assessment, referral for emergencies including severe malaria, IPTp administration, prescribing, laboratory diagnostics, and inventory management. To foster team-based learning, district-level mentors used an integrated, module-based approach to impart both knowledge and practical clinical skills to train the CHOs on management of uncomplicated malaria, diagnosis with mRDTs, pre-referral management and referral of severe malaria cases, malaria in pregnancy, stock management and ordering for resupply, and data management. As part of audit-with-feedback for quality improvement, CHOs were asked to identify possible reasons for performance gaps documented in the facility scorecard. Based on their answers, CHOs used plan-do-study-act cycles to develop action plans to put into place in their CHPS compounds to address gaps in service quality. CHOs were also encouraged to transfer knowledge and skills developed during the internship to other CHPS staff. Before and immediately upon concluding the internship, mentors administered a closed-book questionnaire online via Google Forms while in the classroom setting to assess interns' knowledge (Supplement). Mentors also conducted skills assessments using an observed structured clinical examination (OSCE) approach, using direct observation of CHOs consulting with real patients to evaluate clinical skills and documenting their observations in a Google Form online to track interns' progress (Supplement).^32^ Mentors were given a lunch allowance while they led the internship to offset their opportunity cost; CHOs were given a transportation allowance to permit their travel each day from the CHPS to the referral facility where the training was held.

After the internship, mentors provided continuous on-demand support via phone and WhatsApp; mentors also provided CHOs feedback on referrals made to the district referral hospital. Three months post-internship, the malaria focal person, medical officer, midwife, and health information officer from the district-level mentor teams conducted a follow-up visit to each CHPS compound to reinforce competencies and assess retention of knowledge and skills. This 1-day mentoring visit to each CHO intern's CHPS compound allowed mentor teams to assess provider competencies using the same OSCE assessment as during the internship. Mentors also provided continuous mentoring and supportive supervision and assessed how action plans were being implemented, including reviewing progress on chosen indicators, using a post-training mentoring tool (Supplement). The 3-month post-internship mentoring visit involved all staff at each CHPS compound (including those who had not completed the internship) to encourage a team-based approach to improving service delivery.

## CHO INTERNSHIP PROGRAM ASSESSMENT

**FIGURE 3:**
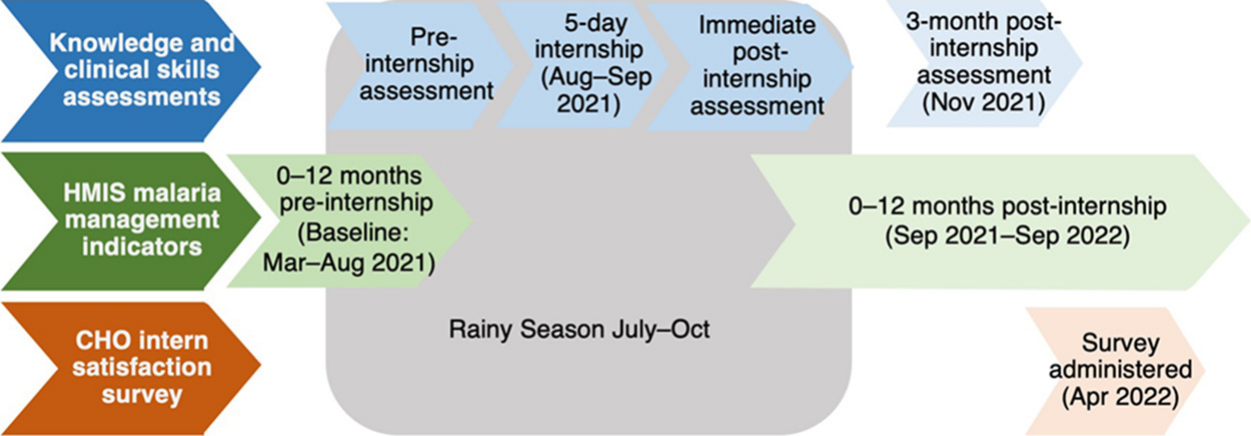
Study Design and Timeline of the Impact Malaria Community Health Officer Internship and Mentoring Program in Ghana Abbreviations: CHO, community health officer; HMIS, health management information system.

We aimed to assess how the CHO internship and subsequent mentoring visit were associated with interns' knowledge and specific clinical skills pertaining to malaria prevention and management over time, as well as changes in treatment outcomes after the internship. To that end, we conducted a mixed-methods before-after study including all 520 participating CHPS compounds (Figure 3) that included (1) a comparison of interns' knowledge and clinical assessment scores before, immediately after, and 3 months post-internship; (2) an analysis of HMIS malaria management indicators 0–12 months post-internship compared to baseline (0–12 months before the internship); and (3) a qualitative CHO intern satisfaction survey.

### Acquisition and Retention of Knowledge and Skills

We compared CHOs' knowledge and OSCE scores on management of febrile illness, severe malaria pre-referral management, and prompt and appropriate referral for severe malaria at 3 points: at the start of the internship (August 2021), immediately post-internship (September 2021), and 3 months after the internship during the post-internship mentoring visit (December 2021). Interns used Google Forms to complete all knowledge assessments. Mentors used the KoboCollect App to record OSCE assessment data during the internship and during the post-internship mentorship visit in December 2021. We stored all data in the Impact Malaria training database. To assess changes in knowledge and competencies associated with the internship, we compared knowledge and OSCE assessment scores before the internship to scores immediately post-internship. To assess retention of competencies over time, we compared knowledge and OSCE assessment scores immediately post-internship to 3 months post-internship.

### Effect on Malaria Management

To determine whether knowledge and skill improvements among CHOs translated into improved clinical practices in malaria management service delivery at the CHPS compounds, we assessed HMIS data on indicators related to malaria management service delivery among all intervention CHPS compounds and a matched group of 520 comparison CHPS compounds in the same regions that did not have a CHO intern or receive mentoring. The comparison CHPS compounds were selected using the same criteria as described in Figure 1. Specific indicators included testing rates for suspected uncomplicated malaria, suspected malaria cases that tested negative but were still treated as malaria (nonadherence to a negative test result), IPTp3+ coverage, pre-referral treatment (for children aged younger than 5 years), proportions of cases referred, and confirmed cases treated with ACTs correctly. HMIS data elements and definitions of these indicators are provided in Supplement Tables S1 and S2. Impact Malaria used quantitative facility-level service data, reported monthly to the national HMIS, for both intervention and comparison facilities. Service statistics reported by CHPS compounds 0–12 months pre-internship (September 2020–August 2021) served as a baseline, and the study team compared this to data reported 0–12 months post-internship (September 2021–August 2022) for each site.

### Participant Experience of the CHO Internship

The study included a post-internship online satisfaction survey sent via an emailed Google Forms survey link to a convenience sample of 120 CHO interns in April 2022. The sample was selected during an adaptive management after-action review with inputs from regional malaria focal persons; the selection strategy was designed to ensure geographic representation from all districts in each region. Facilities were randomly selected, but inclusion criteria included smartphone ownership and Internet access, which limited the sample. The survey elicited interns' experiences and perspectives, inquiring about CHOs' experience with the internship, how it differed from other training methods, and how they were able to translate knowledge and skills gained into practice, including how the internship had affected CHO service provision at the CHPS compound. Survey responses shed light on how CHOs perceived that the internship had affected their competence and knowledge in management of malaria, febrile illness, and pre-referral treatment, as well as actions CHOs had taken since the internship to improve quality of care and translate their training into practice. The study team considered these qualitative accounts of participant and stakeholder experiences with the internship and mentorship alongside quantitative assessment scores to holistically evaluate the internship.

### Data Analysis

#### Knowledge and Skill Retention

A 2-sample t-test at 5% level of significance detected differences in individuals' pre- and post-internship assessment scores as well as differences between assessment scores immediately post-internship and 3 months post-internship.

#### Effect on Service Delivery

A paired t-test using Stata version 14.2 identified statistically significant changes over time in routine monthly HMIS indicators on quality malaria case management (comparing 0–12 months pre-internship to 0–12 months post-internship).^30^ The analysis measured changes in presumptive treatment (treating suspected malaria cases without testing), nonadherence to negative test results, testing for suspected uncomplicated malaria, and IPTp3+ coverage after the internship.

#### CHO Intern Participant Experience Survey

The study team read through survey responses and highlighted emergent themes individually using a thematic analysis approach in Atlas.ti version 7.5. The team then collated and reached consensus on themes through team discussions.

### Ethical Approval

The study obtained a non-human subject research determination from the Johns Hopkins Bloomberg School of Public Health Institutional Review Board, Maryland, USA (IRB 21543). All study participants gave their informed consent.

## RESULTS

### CHO Internship Participant Profile

A total of 281 district-level mentors, including 104 hospital medical officers and 177 individuals from district governments, participated in the internship program; 520 CHOs completed the internship. No mentors or CHOs dropped out; however, 24 of the 281 mentors either were transferred or took leave and were not present for the entire internship period. Each district had 2 or more trained mentors, so internship training activities were not affected by this turnover and attrition. Sixty-four percent of mentors were female, and 49% of interns were female. The most common CHO intern cadre was enrolled nurses (Table 1).

**TABLE 1.** Characteristics of Mentors and Community Health Officers in the Impact Malaria Internship Program in Ghana

	Mentors, No. (%) (N=281)	Community Health Officers, No. (%) (N=520)
Age group, years		
20–29	0 (0.0)	228 (43.8)
30–39	28 (10.0)	268 (51.5)
40–49	168 (59.8)	24 (4.7)
50–59	85 (30.2)	0 (0)
Sex		
Female	180 (64.1)	255 (49.0)
Male	101 (35.9)	265 (51.0)
Cadre		
Medical doctor	73 (26.0)	--
Health information officer	52 (18.5)	--
Medical laboratory scientist	52 (18.5)	--
Malaria focal person	52 (18.5)	--
Midwife	52 (18.5)	78 (15.0)
Enrolled nurse	--	218 (41.9)
Community health nurse	--	146 (28.1)
Nurse	--	78 (15.0)
Years of experience		
<1	0 (0)	117 (22.5)
1–5	0 (0)	292 (56.1)
6–10	84 (29.9)	44 (8.5)
11+	197 (70.1)	67 (12.9)

### Cost

The total cost per CHO trained—inclusive of mentor orientation and training, the 5-day CHO internship, follow-up visits, training materials, transportation allowances for CHOs and mentor teams, and lunch allowance for mentors during the internship—was US$260 per CHO.

### Knowledge and Skills Improvement and Retention

CHOs' knowledge and retention of clinical skills improved across multiple technical areas during the internship and 3 months after the internship compared to immediately after the internship. Over the course of the internship, improvements were identified in clinical competence in patient assessment/history taking (+12.0 percentage points; 95% confidence interval [CI]=8.3, 15.1; *P*<.001); severe malaria assessment and referral (+32.0 percentage points; 95% CI=28.2, 35.8; *P*<.001); fever assessment (+24.9 percentage points; 95% CI=20.9, 29.3; *P*<.001); and knowledge assessment (+15.8 percentage points; 95% CI=10.0, 21.3; *P*<.001*)*. All other technical areas assessed also improved during the internship at the *P*<.001 level of significance (Table 2).

**TABLE 2.** Effect of Internship on Malaria Clinical Competence and Skill Retention for Community Health Officers in Ghana at 3-Month Post-Internship Mentoring Assessment

Clinical Competence Assessment	Pre-Internship Assessment Score Percentage, Mean [SE]	Post-Internship Assessment Score Percentage, Mean [SE]	PP Difference Before-After Internship, Mean (95% CI)	*P* Value	3-Month Post-Internship Score Percentage, Mean [SE]	PP Difference From Internship End to 3-Month Post-Internship, Mean (95% CI)	*P* Value
Patient assessment/history taking	86.2 (2.8)	98.2 (1.2)	+12.0 (8.3, 15.1)^a^	<.001^a^	98.0 (1.0)	+0.2 (−1.1, 2.1)	.642
Severe malaria assessment and referral	44.0 (1.7)	76.0 (1.4)	+32.0 (28.2, 35.8)^a^	<.001^a^	75.1 (1.5)	−1.1 (−5.3, 3.4)	.681
Assessment of fever	56.9 (1.5)	81.8 (1.2)	+24.9 (20.9, 29.3)^a^	<.001^a^	80.0 (1.0)	−1.8 (−5.3, 1.1)	.173
Acute respiratory infection	41.1 (1.7)	71.1 (1.4)	+30.0 (24.6, 34.0)^a^	<.001^a^	65.2 (1.0)	−5.9 (−1.9, −8.8)^a^	.003^a^
Diarrhea	28.0 (1.0)	47.0 (0.9)	+19.0 (16.2, 22.3)^a^	<.001^a^	76.0 (1.0)	+28.0 (24.6, 32.3)^a^	<.001^a^
Medical history	55.9 (1.9)	81.9 (1.5)	+26.0 (22.1, 31.4)^a^	<.001^a^	79.1 (1.4)	-2.8 (−7.1, 1.2)	.136
Knowledge assessment	57.4 (2.8)	73.2 (0.8)	+15.8 (10.0, 21.3)^a^	<.001^a^	-	-	-

Abbreviations: CI, confidence interval; PP, percentage point; SE, standard error.

a Statistically significant difference.

CHOs' knowledge and retention of clinical skills improved across multiple technical areas during the internship and 3 months after the internship compared to immediately after the internship.

Three months after the internship, CHOs retained the same level of competency in history taking, severe malaria assessment and referral, and fever assessment (*P*<.05) (Table 2). Additionally, at this 3-month follow-up, CHO competence in managing diarrhea, which had increased by 19.0 percentage points during the internship, rose by an additional 28.0 percentage points, but competence in managing acute respiratory infections—which had increased by 30.0 percentage points during the internship—declined slightly, by 5.9 percentage points (*P*<.05) (Table 2).

### Malaria Management HMIS Indicators

HMIS data suggest that participation in the CHO internship program was associated with a significant 2.2 percentage point reduction in presumptive treatment during the 12 months post-internship (95% CI=−4.9, −1.5; *P*<.001) (Table 3). IPTp3+ coverage increased significantly in both intervention and comparison CHPS compounds but increased more in intervention CHPS compounds than in comparison CHPS compounds (13.0 percentage points vs. 9.8 percentage points, respectively; *P*<.001). Testing rates for uncomplicated malaria rose from 94.7% to 97.3% (*P*<.001), and pre-referral treatment (for children aged younger than 5 years) significantly improved in intervention CHPS compounds but not comparison CHPS compounds. The percentage of cases referred decreased from 44.3% to 34.3% in intervention CHPS compounds (*P*=.007), compared with a nonsignificant decrease from 48.1% to 47.2% in comparison CHPS compounds (*P*=.294). Treatment correctness did not change significantly in either group.

**TABLE 3.** Comparison of HMIS Malaria Management Indicators in Intervention CHPS Compound Versus Comparison Compound 0–12 Months Before CHO Internship Versus 0–12 Months Post-Internship

	Intervention CHPS Compound (CHO Internship)				Comparison CHPS Compound			
National HMIS Indicator	Baseline^a^ Mean, % (SD)	Post-Intervention^b^ Mean, % (SD)	PP Difference (95% CI)	*P* Value	Baseline^a^ Mean, % (SD)	Post-Intervention^b^ Mean, % (SD)	PP Difference (95% CI)	*P* Value
Presumptive treatment	4.3 (0.0022)^c^ N=111,471	2.1 (0.0012)^c^ N=63.322	−2.2 (−4.9, −1.5)^c^	.001^c^	3.4 (0.0165) N=70,749	2.9 (0.0204) N=62,758	+0.4 (−3.4, 2.2)	.239
Nonadherence to negative test results	0.9 (0.0022)^c^ N=21,053	0.7 (0.0019)^c^ N=16,830	−0.2 (−4.8, −1.1)^c^	.009^c^	1.3 (0.0057) N=24,980	0.8 (0.0054) N=17,477	−0.5 (−2.5, −0.1)	.015
IPTp3+ coverage	45.7 (0.0891)^c^ N=101,672	58.7 (0.0670)^c^ N=138,440	+13.0 (6.4, 18.7)^c^	.000^c^	48.1 (0.0961)^c^ N=105,972	57.9 (0.0450)^c^ N=134,442	+9.8 (+4.5, +14.6)^c^	.000^c^
Testing rate for suspected uncomplicated malaria	94.7 (0.0234)^c^ N=2,292,065	97.3 (0.0130)^c^ N=2,471,574	+2.6 (+1.1, +6.4)^c^	.000^c^	95.9 (0.0130) N=2,009,378	96.5 (0.0241) N=2,142,251	+0.5 (−3.4, +2.3)	.187
Percentage of confirmed cases referred	44.3 (0.3210)^c^ N=33,430	34.3 (0.291)^c^ N=32,100	−10.0 (−1.8, −18.0)^c^	.007^c^	48.1 (0.4192) N=21,083	47.2 (0.6202) N=27,237	−1 (−5.2, +4.1)	.294
Appropriate pre-referral treatment (aged<5 years)	64.7 (0.0031)^c^ N=10,208	79.8 (0.0562)^c^ N=9,833	+15.1 (+7.1, 22.7)^c^	.000^c^	70.0 (0.0467) N=11,304	72.3 (0.0762) N=11,874	+2 (−2.1, +4.3)	.096
Confirmed malaria cases treated with ACTs	95.0 (0.1087) N=536,572	100.0 (0.0464) N=1,097,219	+5.0 (−3.8, +13.1)	.090	88.1 (0.0235) N=657,432	92.4 (0.2102) N=945,652	+4 (−2.3, −9.0)	.186

Abbreviations: ACT, artemisinin-based combination therapy; CHO, community health officer; CHPS, Community-based Health Planning and Services; CI, confidence interval; HMIS, health management information system; IPTp3+, intermittent preventive treatment of malaria for pregnant women, 3+ doses; N, numerator of each indicator; PP, percentage point; SD, standard deviation.

a 0–12 months before internship.

b 0–12 months post-internship.

c Statistically significant associations.

### Intern Satisfaction and Experiences

Of the 120 CHOs surveyed, 107 responded (89.1%). Fifty-seven (53.2%) were female, and approximately half (51.4%) were aged 30–39 years. More than half (57.0%) had 1–5 years of work experience. Thematic analysis of survey responses highlighted how their experience of the CHO internship program had differed from previous experiences and how they had translated their new skills into practice.

Interns widely concurred that being trained at their assigned district referral hospital under the mentorship of a medical officer mentor was a valuable experience because it built their familiarity with district referral hospital health workers and the various hospital departments in a way that respected their value as critical points of care in the community. CHOs reported that the internship differed in quality and utility from previous training they had received because the learning experience was very interactive and involved practice with real patients with mentor support. CHOs appreciated the opportunity to share their challenges with their mentors and perceived that their experiences built their confidence in conducting physical examinations, taking histories, probing for differential diagnosis of fever, administering pre-referral treatment, and making referrals, as well as in developing useful skills such as completing inventory control cards and proper register and reporting documentation.

*The training gave us the opportunity to have one-on-one encounters with the mentors … to share our challenges … it has built our confidence level in carrying out our duties. In other trainings, they didn't give us the chance to assess ourselves on what was taught.* —Intern, Bono region

While interns mentioned that CHPS compounds occasionally experienced stock-outs of malaria diagnostic and treatment supplies, mentors provided prompt training on how to update commodity inventory cards, which helped ensure requests for additional supplies were made in a timely fashion to prevent stock-outs.

Interns noted that their mentors effectively met the needs of individual learners. For example, if an intern performed a procedure incorrectly, mentors provided individual assistance to ensure that the intern could correctly and confidently perform the skill before the end of the training session. Feeling confident in their skills was rewarding and motivating for participants.

*Consulting was a big challenge for me but I was able to do it successfully without difficulty after the internship.* —Intern, Western North region

Interns felt that the internship had improved their knowledge, competence, and confidence regarding testing and treatment, which they felt had increased their adherence to the WHO test, treat, and track policy; adherence to treating only patients with a positive test result; and prompt referral after pre-referral treatment.

*We are prompt in referring unlike before where we will wait for quite a longer time before thinking of referring, which leads to severe condition.* —Intern, Greater Accra region

*Once the danger signs in certain systems of the body are noticed, prompt referral is initiated.* —Intern, Oti region

*Severe cases are promptly referred as per national protocols.* —Intern, Bono East region

Interns reported that their newfound knowledge helped them improve community education during home visits and community *durbar* (festivals), which have reduced the malaria burden at the community level.

*About 40% of the total cases treated were malaria, but that has been reduced to about 27% after the training, because of proper assessment of cases in the management of febrile illness.* —Intern, Bono region

*[A]fter the training, much information was gained on the prevention of malaria, which was transferred to the communities and has helped in reduction of malaria cases in our community …* —Intern, Volta region

Nearly all participants reported that they had seen reductions in severe cases, improved pre-referral management of severe malaria cases, and more prompt referrals for severe malaria. Participants credited these changes to prompt, high-quality management of uncomplicated malaria cases in their facilities that prevented progression to severe malaria. Interns viewed these outcomes as a direct outgrowth of confidence and skills gained from the CHO internship.

## LESSONS LEARNED

Providing a group training experience at the district referral hospital, reinforced through a mentoring visit to CHPS compounds to support and evaluate CHOs several months post-internship, led to observed improvements in provider knowledge and competence around malaria case management and diagnosis and treatment of febrile illnesses. The internship strengthened the capacity of CHOs for quality malaria service delivery: interns retained competency gains for most skills measured via OSCE several months after the conclusion of the internship.

Additionally, improvements observed in several HMIS malaria management indicators (presumptive treatment, IPTp3+ coverage, and testing rate) from baseline to 6 months following the internship suggest that the CHO internship may have contributed to meaningful improvements in malaria management practice, as well as potential cascade effects to other staff at CHPS compounds. Participants in the internship were universally positive about how the internship had improved their knowledge, clinical skills, and confidence. Most believed their improved practice was associated with better outcomes for their clients. An after-action review with mentors after the program showed that they were pleased with the balance of hands-on clinical opportunities with lectures and discussions and felt these training opportunities effectively addressed gaps in CHO knowledge and skills. They suggested that the 5-day internship was too brief for the content taught and recommended a 2-week internship period.

A novel feature of the CHO internship was its explicit focus on connecting district referral facilities with CHPS compounds in their catchment areas by building supportive mentor-intern relationships. The proportion of cases referred decreased after the intervention, which aligns with interns' beliefs that they were more capable of handling uncomplicated cases at the CHPS level; delays in treating uncomplicated malaria are linked to progression to severe disease.^33^ In many countries, delayed or incomplete referrals (particularly if pre-referral treatment is administered) are key barriers to quality malaria management practices. Although these delays can originate at the household level, many deaths from severe malaria are associated with health workers at referring facilities who do not provide prompt referral and pre-referral treatment.^34^ However, to date, malaria case management interventions have tended to recommend improving and monitoring providers' adherence to malaria referral guidelines rather than strengthening referral relationships.^12^^,^^35^^,^^36^ Building the internship around a mentorship model encouraged positive rapport between CHOs at community-based primary care facilities and providers at their referral facilities; CHO interns also benefited from increased familiarity with the system, procedures, and personnel at the district referral hospital to which they referred cases. Additionally, after the internship, referral contacts at the district referral hospital were clearly posted in each CHPS compound, which helped to formalize these relationships. While some mentors were transferred or took leave during the intervention period, training multiple mentors per district helped to minimize disruption to mentoring activities. Through mentor-intern relationships and the mentoring visits paid to CHPS compounds, mentors from the district level could appreciate the multiple challenges faced by providers at poorly performing facilities in the prompt referral and appropriate pre-referral treatment and stabilization of severely ill patients, improving empathy and enhancing opportunities for mentor support.

Importantly, improvements in CHO competencies were not retained in all technical areas, likely due to insufficient numbers of particular types of cases presenting to CHPS compounds during the post-internship period. The internship took place in the middle of the rainy season (July to October), and the follow-up visit was during the dry season, so seasonality may have played a role. Retention of these and other key knowledge areas and clinical skills could wane over time. Continued mentoring and supportive supervision through mentoring visits, regular assessments, and refresher trainings as appropriate could address areas where competency declines over time while fostering strong referral relationships through mentor-intern relationships. Facility-based mentors offer continued on-demand mentoring via phone and WhatsApp, but in-person clinical mentoring visits might better sustain clinical skills and knowledge. Modeled on the promising results and approach of the CHO internship, Impact Malaria has been working with the district health management team to develop a subdistrict supportive supervision tool and process to be used by the subdistrict head to encourage higher quality supportive supervision for malaria at CHPS compounds in the future.

Continued mentoring and supportive supervision could address areas where competency declines over time, while fostering strong referral relationships through mentor-intern relationships.

The acceptability and low cost of the CHO internship program have led to its adaptation and planned inclusion in Ghana's National Malaria Control Strategic Plan 2021–2025 as the national model for training CHOs, and the National Malaria Control Programme is considering lessons from the after-action review with mentors as plans for scale-up progress. Additional domestic funding and resources will be needed to equip CHOs in Ghana for this phase. Lessons from the successful implementation of the CHO internship program in Ghana are potentially applicable to other technical areas and other countries where improvements in provider competence and referral relationships are needed. Competing demands on staff at higher-level facilities may complicate the feasibility and/or sustainability of ongoing supervision and mentoring of CHO. However, quality improvement at primary care facilities ultimately reduces patient burden and improves outcomes at referral facilities, incentivizing efforts to foster effective relationships through mentoring. Future programs should consider the feasibility of establishing similar internships for other community-based health cadres beyond CHOs, including drug vendors, pharmacists, laboratory technicians, and community health workers, as such efforts could improve their adherence to treatment and referral. Although CHO interns perceived that case management and patient outcomes had improved as a result of their internship and mentoring visits, future research should build on these findings by testing the impact of various internship formats and mentoring models on clinical outcomes, especially those related to referral.

### Limitations

Triangulation using a mixed-methods approach and multiple data sources demonstrated clear improvements in provider performance, low cost, and high acceptability of the intervention. More rigorous methodologies than our pre-post assessment of program learning for quality improvement could more accurately assess the impact of the internship and ongoing mentoring on malaria outcomes. Our design is subject to several potential sources of bias, including social desirability and cognitive bias, as the intern satisfaction survey was based on their perceptions and self-report, and reporting bias if the mentors conducting performance assessments for CHOs they mentored consciously or subconsciously felt motivated to inflate CHOs' OSCE scores. However, mentors and CHOs did not receive any incentives that would increase the likelihood of these biases. Ongoing malaria prevention activities in several of the study regions (routine insecticide-treated bed net distribution in all regions and seasonal malaria chemoprevention during the rainy season in Savannah, Northern, and North East regions) could be unmeasured confounders. These activities were unlikely to affect most of the key HMIS indicators we measured, except that decreased cases of uncomplicated and severe malaria during peak transmission season due to seasonal malaria chemoprevention may have decreased severe cases among children aged younger than 5 years. Seasonality of malaria transmission could affect the number of cases. By analyzing HMIS indicators for 12 months before and 12 months after the intervention, we hoped to minimize any effects of seasonality. Stock-outs of RDTs or sulfadoxine-pyrimethamine could also affect the diagnosis and treatment of malaria, but we did not observe fluctuations in testing rates indicative of procurement problems. Last, it is possible that mentoring could have affected routine supportive supervision frequency and content from the district, which could also affect the quality of care. However, the independence of these pathways (supportive supervision visits come from the subdistrict head with funding from the national level and mentoring from referral hospitals) renders this unlikely.

When triangulated with improvements in knowledge and practices, as well as intern satisfaction, improvements at intervention CHPS compounds that were not seen at comparison CHPS compounds—particularly for testing of uncomplicated malaria and referral—lends support for the usefulness of mentorship as a tool that may improve case management of malaria. Future longitudinal studies are needed to assess the effect of ongoing mentorship on referral practices and outcomes and confirm improvement in HMIS malaria indicators.

## CONCLUSION

Participation in an intensive internship designed to strengthen malaria prevention, clinical case management, and referral skills—reinforced via mentoring relationships with the district referral hospital and district health management team—led to clinically meaningful improvements in CHO knowledge, competence, and malaria case management behaviors in Ghana. These improvements were retained several months after the conclusion of the internship, and participants were universally positive about their experiences and the impact of the internship on the quality of the malaria care services they and other staff provided at their CHPS compound. Establishing strong, supportive relationships between trained primary health care workers like CHOs and mentors at referral facilities is an underused strategy to improve quality of care, reduce delays and problems with referrals, and maximize application of local clinical expertise. Improving the quality of malaria diagnosis, treatment, and referral that CHOs provide could reduce malaria morbidity and mortality, especially among pregnant women and young children in Ghana. Potential applications of this strategy extend far beyond malaria case management and beyond Ghana, holding promise to extend the reach and effectiveness of primary health care in many low- and middle-income countries.

## Supplementary Material

GHSP-D-23-00050-Supplements.pdf

## Notes

**Peer Reviewed**

**First published online:** November 3, 2023.
