# Supplementary material for: Improving Malaria Case Management and Referral Relationships at the Primary Care Level in Ghana: Evaluation of a Quality Assurance Internship
Source: Glob Health Sci Pract. 2023 Dec 22;11(6):e2300050. doi: 10.9745/GHSP-D-23-00050 (PMC10749655; doi:10.9745/GHSP-D-23-00050)
Supplement: GHSP-D-23-00050-Supplements.pdf [file GHSP-D-23-00050-Supplements.pdf]

Supplement to: Asiedu A, Haws R, Gyasi A, et al. Improving malaria case management and referral relationships at the primary care level in Ghana: evaluation of a quality assurance internship. *Glob Health Sci Pract*. 2023;11(6):e2300050. https://doi.org/10.9745/GHSP-D-23-00050

Supplement Files

Pre-Course Assessment

Competency Checklist

Post-Training Mentoring Tool

Table S1. HMIS Data Elements and Definitions

Table S2. Malaria Indicators Using HMIS Data Elements

PRE-COURSE ASSESSMENT

CHO INTERNSHIP TRAINING

Name:………………………………… Facility Name:……………………………………….

Date: Cadre:

INSTRUCTIONS: Please select ONE best answer ONLY for each question

Questions:

1. The following three are common symptoms of uncomplicated malaria.
   a. Fever, headache, body pains
   b. Fever, cough, convulsion
   c. Headache confusion, vomiting
   d. Fever, vomiting everything, refusing to eat

2. The following are recommended medicines or combination of medicines for the treatment of uncomplicated malaria in Ghana.
   a. Sulphadoxine-Pyrimethamine
   b. Artesunate-Amodiaquine
   c. Artemether- Lumefantrine
   d. Dihydroartemisin-Pipepraquine
   e. Quinine-Clindamycin Combination
   f. All above
   g. All except A.
3. The medicines indicated below can be used for treatment in case of malaria treatment failure for uncomplicated malaria?
   a. Quinine Injection
   b. Artemether Injection
   c. Artesunate Injection
   d. Any of the ACTs other than the one used for the initial treatment
   e. Artesunate suppository
4. The following defines severe malaria?
   a. Malaria with no vital organ failure
   b. Medical emergency with plasmodium malariae present in the blood
   c. Any Febrile illness with vital organ failure
   d. Confirmed malaria with life threatening complication(s)
   e. Confirmed malaria with more than two life threatening complication(s)
5. The following group(s) of people in the population are most likely to develop severe malaria?
   a. People in rural areas

b. Children under below five years of age
c. Pregnant women
d. Travelers from non-endemic areas
e. a,b,c
f. b,c,d
g. a,b,c,d

6. The following disease condition does not present with symptoms which are similar to uncomplicated malaria especially in children?
   a. Acute Respiratory tract infections
   b. Urinary Tract Infections
   c. Otitis Media
   d. Typhoid Fever
   e. Asthma

7. These factors or reasons account for why people with malaria may die?
   a. Delay in seeking treatment
   b. Poor or incomplete treatment leading to severe malaria
   c. Financial challenges
   d. Use of non-recommended or ineffective medicines
   e. a and b only
   f. b and d only
   g. a,b,c,d

8. The following steps are used to manage a child below 5 years who has a febrile illness
   a. Take a good history to exclude all possible causes of fever
   b. Take a good history and give anti-malaria medicine
   c. If the child has fever and malaria with a life threatening condition is suspected, manage as uncomplicated malaria
   d. a and d only
   e. If you suspect malaria, do a test, treat and track
   f. All Above

9. The following are all common complications of Severe Malaria
   a. Fever, Loss of appetite, convulsion, headache, anaemia
   b. Hypoglycaemia, severe anaemia, convulsions, Jaundice, splenomegaly
   c. Hypoglycaemia, severe anaemia, convulsions, haemoglobinuria, difficulty in breathing
   d. Difficulty in breathing, severe anaemia, general body pains, headache convulsion

10. The 5 “General Danger Signs” in a sick child include

a. Loss of appetite, vomiting everything, history of convulsion in current illness, convulsion now, lethargic/unconscious
b. Not able to feed or breastfeed, difficulty in breathing, history of convulsion in current illness, convulsion now, lethargic/unconscious
c. Not able to feed or breastfeed, vomiting everything, severe dehydration, convulsion now, lethargic/unconscious
d. Not able to feed or breastfeed, vomiting everything, history of convulsion in current illness, convulsion now, lethargic/unconscious

11. The following is true of parenteral anti-malaria medicines used in the management of severe malaria:
a. Injection artesunate b. Injection Quinine c. Rectal Artesunate, d. Dihydroartemisinin-Piperaquine e. Inj Artemether
    a. a only
    b. b only
    c. a and c only
    d. a, b and e only
    e. All
12. The following is true of parenteral anti-malaria medicines used in the management of severe malaria in the first trimester of pregnancy:
a. Injection Artesunate b. Injection Quinine c. Rectal Artesunate, d. Dihydroartemisinin Piperaquine e. Inj. Artemether
    a. a and b only
    b. a only
    c. b only
    d. a, b, e only
    e. all
13. The following is true of parenteral anti-malaria medicines used in the management of severe malaria in the second and third trimesters of pregnancy:
a. Injection Artesunate b. Injection Quinine c. Rectal Artesunate, d. Dihydroartemisinin Piperaquine e. Inj. Artemether
    a. a only
    b. b only
    c. a and c only
    d. a, b and e only
    e. all
14. The Rectal artesunate is indicated as pre-referral medicine for severe malaria in which age category of patients
    a. All patients
    b. Children below 5 years
    c. Children below 6 years or weight < 25 kg

d. Pregnant women only
e. Patients above 6 years only

15. The recommended dose and the schedule for Inj. Artesunate for the management of severe malaria in a child less than 20 kg is
    a. 2.4mg/kg body weight at time 0, 12 hours, 24 hours then every 24hrs for up to 7 days
    b. 3.0 mg/kg body weight at time 0 and 24 hours then every 24hrs for up to 7 days
    c. 2.4mg/kg body weight at time 0, 12 hours, 24 hours then every 12hrs for up to 7 days
    d. 3.0mg/kg body weight at time 0, 12 hours, 24 hours then every 24hrs for up to 7 days
    e. 3.0 mg/kg body weight at time 0, 12 hours, 24 hours then every 12 hours for up to 7 days
16. The recommended dose and the schedule for Injection Artesunate for the management of severe malaria for a patient ≥ 20 kg is
    a. 2.4mg/kg body weight at time 0, 12 hours, 24 hours then every 24hrs for up to 7 days
    b. 3.0 mg/kg body weight at time 0, 24 hours then every 24hrs for up to 7 days
    c. 2.4mg/kg body weight at time 0, 12 hours, 24 hours then every 12hrs for up to 7 days
    d. 3.0mg/kg body weight at time 0, 12 hours, 24 hours then every 24hrs for up to 7 days
    e. 2.4 mg/kg body weight at time 0, 12 hours, 24 hours then every 24hrs for up to 5 days
17. How long can reconstituted and diluted Artesunate for injection be stored?
    a. 30 minutes
    b. 60 minutes
    c. Up to 4 hours
    d. 24 hours
    e. No time restriction as long as it is stored in a fridge
18. How many hours after the last parenteral Artesunate dose should the first oral dose follow on treatment be given?
    a. After 12 hours up to 24 hours
    b. Between 4 and 12 hours
    c. At 24 hours
    d. Any time after 24 hours
19. In a child with severe malaria with convulsion, after assessing and securing the airway, the next most important actions is to
    a. Stop convulsion by administering anti-convulsant
    b. Tepid sponge the patient
    c. Give 1st dose of recommended anti-malaria
    d. Check blood glucose and first manage for hypoglycaemia if hypoglycaemic

e. Re-assure the care taker

20. Dosage regimen for Parenteral Diazepam for managing convulsion in children is
    a. IV Diazepam 0.25mg/kg (maximum 10mg per dose) to be repeated up to 1 dose
    b. IV Diazepam 0.5mg/kg (maximum 10mg per dose) to be repeated up to 1 dose
    c. IV Diazepam 10mg stat repeat prn
    d. IV Diazepam 0.1mg/kg stat
21. What is the threshold of Blood Glucose that requires intervention?
    a. Blood Glucose < 5.0 mmol/l
    b. Blood Glucose < 3.0 mmol/l
    c. Blood Glucose < 2.2 mmol/l
    d. Blood Glucose <4.0 mmol/l
22. The following describes how hypoglycaemia is managed
    a. 10% Dextrose at 10mls/kg bolus
    b. 5% Dextrose at 2 – 4mls/kg bolus; follow-up with IV Infusion containing 5 – 10% dextrose at 5ml/kg per hour.
    c. 10% Dextrose at 2 – 4mls/kg bolus, Follow-up with IV Infusion containing 5 – 10% dextrose at 5ml/kg per hour
    d. 15% Dextrose at 2 – 4mls/kg bolus; Follow-up with IV Infusion containing 5 – 10% dextrose at 5ml/kg per hour

COMMUNITY HEALTH OFFICER ATTACHMENT PROGRAMME

Checklist to assess competency of lower level health staff in febrile case management

Common illnesses to assess: malaria, respiratory tract infections, gastrointestinal infections, Ear nose and throat infections, mouth and gum disorders, urinary tract infections and sexually transmitted infections and skin infections

REGION…………………………………………………………….DISTRICT…………………………………………………………….

CHO attachment Health facility……………………………………………Facility type: Hospital [ ] Polyclinic [ ] Health Centre [ ]

Name of Observer ……………………………………………………… Designation ………………………………………………………

Contact number and email: ……………………………………………………………………………………………………

Details of health worker undertaking attachment in febrile case management

**Health facility (s)he works**						
**FACILITY TYPE**	CHPS	CLINIC	MATERNITY HOME	HEALTH CENTRE	POLYCLINIC	HOSPITAL
**FACILITY OWNERSHIP**	QUASI-GOVT	GOVT		MISSION (CHAG)	PRIVATE	OTHER
**NAME OF HEALTH WORKER**						
**Contact phone and email**						
**CADRE OF STAFF**	CHO/CHN	EN/RN/SRN	PA/MA	MD	MIDWIFE	OTHER

Patient data (Score – 2 for complete)

Age of Patient:…………………………………………………………Sex of Patient:………………………………………Weight: …………………

Assessment

	ASK	LOOK, LISTEN and FEEL	Remarks
**General danger sign or signs of severe disease**	1. Unable to drink or breastfeed<br>2. vomiting everything<br>3. History of convulsion in current illness<br>4. and/or Any symptoms of severe disease<br>a. Dark coloured urine (have look at sample)<br>b. Severe pallor (conjunctiva, palm, lips)<br>c. High temperature (≥37.4 for newborn) and ≥38.5^{0}C)<br>d. Hypothermia (low temperature ≤ 35.5^{0}C especially in newborn<br>e. Fast breathing; chest in-drawing<br>f. Signs of Severe dehydration	1. lethargy or Unconscious<br>2. convulsion now<br>3. and/or Any signs of severe disease	**Score 10 Max**<br>a. Assess General Danger Signs – 5<br>b. Signs of severe disease 5
Fever	History of fever? Yes [ ] No [ ]<br>If yes,<br>- how long? Yes [ ] No [ ]<br>- any other problem? Yes [ ] No [ ]<br>- medications taken? Yes [ ] No [ ]	Ask the health worker what (s)he would look out for or do (tick)?<br>- measure temperature? Yes [ ] No [ ]<br>- lethargy?<br>- confusion/ convulsion?<br>- stiff neck	Score 5 for correct assessment
Acute Respiratory Infections	Did health staff ask of cough or difficulty in breathing? Yes [ ] No [ ]<br>If yes, did health worker ask the following?<br>- for how long? Yes [ ] No [ ]<br>- sputum or no sputum? Yes [ ] No [ ]<br>If productive cough, blood in sputum? Yes [ ] No [ ]<br>- chest pain? Yes [ ] No [ ]<br>- night sweats? Yes [ ] No [ ]<br>- smoking history? Yes [ ] No [ ]<br>- Drug history for any chest infection? Yes [ ] No [ ]<br>Did health worker ask of:	Did health staff look and listen for following?<br>- count breaths in one minute? Yes [ ] No [ ]<br>○ Ask Respiratory rate for fast Breathing (different age groups)<br>- Difficulty in breathing Yes [ ] No [ ]<br>○ wheezing?<br>○ chest indrawing?<br>○ intercostal recession,<br>○ flaring ala nasae, Yes [ ] No [ ]<br>○ Stridor – demonstrate<br>- Count pulse? Yes [ ] No [ ]<br>- Measure BP? Yes [ ] No [ ]	Score 10 for complete assessment

	ASK	LOOK, LISTEN and FEEL	Remarks
	- stuffy/ running nose or catarrh? Yes [ ] No [ ]		
Mouth<br>And<br>ENT	Did health worker ask for the following?<br>- pain in throat Yes [ ] No [ ]<br>- problems swallowing? Yes [ ] No [ ]<br>- problems chewing? Yes [ ] No [ ]	Did health worker look into the mouth of client? Yes [ ] No [ ]<br>If yes, ask health worker to mention what (s)he is looking for in mouth (abnormalities)<br>- tooth problems? Yes [ ] No [ ]<br>- swollen gums? Yes [ ] No [ ]<br>- bleeding from gums? Yes [ ] No [ ]<br>- white patches? Yes [ ] No [ ]<br>- red pharynx? Yes [ ] No [ ]<br>- tonsils (big/ pus)? Yes [ ] No [ ]<br><br>Others<br>- swelling in jaw? Yes [ ] No [ ]<br>- feel for enlarged neck lymph nodes? Yes [ ] No [ ]	Score 10 for complete assessment
	Ear pain or discharge? Yes [ ] No [ ]<br>If yes to, which ear? Yes [ ] No [ ]	Did health worker assess for<br>- pain: by pulling the ear lobe? Yes [ ] No [ ]<br>- discharge? Yes [ ] No [ ]	
Diarrhoea	Did health worker ask of diarrhea? Yes [ ] No [ ]<br>If yes,<br>- how long? Yes [ ] No [ ]<br>- is there an outbreak of diarrhea in client's community? Yes [ ] No [ ]<br>- is client on treatment for diarrhea? Yes [ ] No [ ]<br>Did health worker ask for abdominal pain? Yes [ ] No [ ]<br>Did health worker ask of abdominal swelling? Yes [ ] No [ ]<br>Did health worker ask of blood in stool? Yes [ ] No [ ]	Ask health worker to demonstrate how to assess for dehydration. Were the following assessed correctly<br>- skin pinch? Yes [ ] No [ ]<br>- sunken eyes? Yes [ ] No [ ]<br>- thirst? Yes [ ] No [ ]<br>- Correctly classify the diarrhoea	Score – 10 max

	ASK	LOOK, LISTEN and FEEL	Remarks
Genito-urinary	Does health worker ask of<br>- burning or pain on urination? Yes [ ] No [ ]<br>o If yes, how long? Yes [ ] No [ ]<br>- increased frequency of urination? Yes [ ] No [ ]<br>- flank pain? Yes [ ] No [ ]<br>- sores or swelling in genital or anal area? Yes [ ] No [ ]<br>In females:<br>- abnormal vaginal discharge? Yes [ ] No [ ]<br>- last menstrual period or menses last month? Yes [ ] No [ ]<br>In males:<br>- urethral discharge? Yes [ ] No [ ]<br>- scrotal pain or swelling? Yes [ ] No [ ]<br>In all sexually active patients, sexual history? Yes [ ] No [ ]	Ask health worker, what would you do if one of these symptoms is present?	Score 10
Nutrition status and anaemia	Did health worker Ask<br>- for weight loss? Yes [ ] No [ ]<br>- diet history? Yes [ ] No [ ]<br>- alcohol use? Yes [ ] No [ ]<br>If pallor, did health worker ask<br>- black/ dark stools? Yes [ ] No [ ]<br>- blood in stool? Yes [ ] No [ ]<br>- blood in urine? Yes [ ] No [ ]<br>- in menstruating females, heavy menstrual period? Yes [ ] No [ ]	Did health worker look for/ document<br>- visible wasting?<br>- oedema? Yes [ ] No [ ]<br>- check weight of client? Yes [ ] No [ ]<br><br>Did health worker assess patient for<br>- pallor (palmer/conjunctival)? Yes [ ] No [ ]	Score Max 10
Skin	Did health worker ask of<br>- sores or swelling on skin or other skin problem? Yes [ ] No [ ]<br>- If a sore or swelling, is there discharge? Yes [ ] No [ ]<br>- is there pain? Yes [ ] No [ ]<br>- is client on treatment? Yes [ ] No [ ]	Ask health worker to demonstrate how to assess	Max Score 3

	ASK	LOOK, LISTEN and FEEL	Remarks
Medical history	Medical history<br>- asthma? Yes [ ] No [ ]<br>- hypertension or heart disorder? Yes [ ] No [ ]		Max Score 2
Investigation	Orders appropriate Lab Investigation<br>Fever – RDT for Malaria<br>Anaemia – Hb		Max Score 3
Correctly Diagnosis or Classification of Disease			Score 5

Malaria Case Management Post-Training Mentoring Tool

General Instructions

A. Before visit:

1. Review previous visit findings and recommendations (if any)
2. Review facility Malaria DHIMS Data
3. Notify the facility at least One week before the scheduled visit and remind a day before the visit
4. Make available to the Facility the monitoring tool and all other tools to be used during the visit

B. During the Visit:

1. Entry Interview - The supervisory team should first meet with the Facility Management, Clinical Coordinator and Ward in-charges involved in the Malaria training to discuss the follow-up from the training as well as any system changes that have been implemented since the training or the last visit.

2. Following that group discussion, the team should divide responsibilities, if possible visit the various units participating in the training
   i. Assess facility preparedness for managing malaria
   ii. Review and document process maps prepared
   iii. Review and document PDSA prepared
   iv. Support teams to prepare action plans based on findings

3. The results of the assessment should be shared with the clinic staff at the end.

C. Before Exit

1. Exit Conference with management and Team
2. Agree on action plan and Follow-up Plan

D. Prepare and Submit written technical report to the Facility within a maximum 2 weeks after visit

Facility Details

Region: ……………………………………. District: ……………………………. Facility Name: …………….………..…………………………

Facility Type: …………………………. Facility Ownership: ……………………….. Date of Training: ………..…..………………….…..

Date of First Follow-up visit: ……………………………..…… Date of Last Follow-up visit: ………………..……………………………….

Date of Current visit: ………………………………. Start Time: ………………………..…. End Time: ……………………..……………….…

Units visited: (Tick √ All): 1. OPD 2. Paediatric Ward 3. Maternity Ward 4. Laboratory 5. Pharmacy 6. ANC 7. Male Medical 8. Female Medical 9. District Health Directorate 10. HI Unit 11. Any Other Unit (Specify)…………

A. Supervisors' Details

Name	Position	Contact Number/E-mail

B. Contacts During the Visit

Unit	Contact person	Position	Phone number
Management			
OPD			
Emergency Ward			
Paediatric ward			
Maternity Ward			
Laboratory			
Other unit (specify)			

C. Review facility Malaria DHIMS Data (Pre-visit)

Month	Malaria Admissions	Malaria Deaths	U5 CFR	% Uncomplicated malaria suspected tested	% suspected uncomplicated malaria not tested but treated as malaria	% Confirmed uncomplicated malaria cases treated with ACTs	ANC registrants	No. of Pregnant women given IPTp 1	No. of Pregnant women given IPTp 3	IPTp 1 coverage	IPTp 3 coverage
Jan											
Feb											
Mar											
Apr											
May											
Jun											
Jul											
Aug											
Sep											
Oct											
Nov											
Dec											

D. Assessment of Facility

1. Availability of Treatment Protocols and Wallcharts – Visit follow Service Delivery Points and assess for the following (Indicate Yes/No/Non-Applicable(N/A)

SN	**Job-Aids (Protocols/Charts/Manuals**	OPD	Emergency	Paediatric Ward	Maternity Ward	ANC
1	Flow chart for the diagnosis and treatment of malaria					
2	Artesunate IV/IM administration poster					
3	Artesunate-Lumefantrine Dosing Schedule					
4	Artesunate-Amodiaquine Dosing Schedule					
5	Rectal Artesunate Dosing Schedule					
6	Management of Convulsion					
7	Management of Hypoglycaemia					
8	IPTp-SP Administration Guide					
	**Malaria Indicators Wallcharts**					
1	Outpatient Malaria Indicator Monitoring Chart					
2	ACT Adherence Monitoring Chart					
3	Inpatient Malaria Indicator Monitoring Chart					
4	IPTp and ITN Indicator Monitoring Chart					

2. Availability of Anti-Malaria Medicines and Other Medicines/Items for Malaria Management at the Facility

Medicine	In Stock today (Yes/No/EXPIRED)	Out of stock more than 7 days in past 3 months? (Y/N)	If Yes for how many days?
Inj Artesunate (60mg)			
Inj Other mg Artesunate			
Inj. Artemether			
Inj Quinine			
Tab. Quinine			
Tab. A-L			
Tab. A-A			
Inj Diazepam			
Inj Phenobarb			
10% Dextrose			
5% dextrose			
R/Lactate/N-Saline			
Blood Giving Set			
Blood at Blood Bank			
RDT Test Kit			

3. Availability of Availability of Functional Equipment and health Commodities for Emergency Care (Indicate Yes – Functional; Yes – Non-functional; No; Expired; NA)

COMMODITY	OPD	Em. Ward	Paed Ward	Mat. Ward
Thermometer				
Sphygmomanometer – Adult				
Weighing Scale				
IV Cannula **– Paed**				
IV Cannula **Adult**				
Syringes & Needles				
IV Giving Set				
Glucometer				
Oxygen				
Pulse Oximeter				
Suction Machine				

4. Availability of Laboratory Services (Indicate Yes/No)

**Test**	**Availability**	**Any Comment?**
Hb		
FBC		
Sickling		
Blood film for Malaria Parasites (Thick and Thin)		
Malaria RDT (Indicate points of use and monitoring in comments)		
G-6PD Screen		
Blood Glucose		
LFT		
BUE & Cr		
Blood Grouping and Cross-matching		

E. Assessment, monitoring and management of Severe Malaria. Randomly Select Five Severe malaria cases Folder after April 2019 and assess for the following and score as appropriate (Indicate Yes; Yes – timely and complete; Yes – Incomplete/not timely; no)

No.	Assess for Complications	Temp Timely	Resp. Rate (timely)	Pulse Rate Timely	Treatment Chart (Timely & Complete	Time to initiate treatment after admission (minutes)	Anti-Malaria Used	Anti-malarial Medication Administered Per guidelines	Malaria test Results (Positive, Negative, Not done)	Compliance with malaria Negative test results (yes/No)

F. Review Action Plans Developed by Trainees

1. Are the PDSA prepared by the teams completed and available (document in Table below

Unit	Change Idea Developed	PDSA (Available complete; incomplete; no PDSA	Status of Implementation
OPD			
ER			
Paediatric			
Maternity			
Laboratory			
ANC			

2. Are the action plans (PDSA) developed by the team during the training shared with their immediate Supervisor/Ward in-charge? ☐ Yes ☐No

   a. If no, why?

      i. ……
      ……..

      ii. ……..

      iii. ……..

3. Are the action plans (PDSA) developed by the team during the training shared with other Health Workers in the unit? ☐ Yes ☐No

   a. If no, why?

      i. ……
      ……

      ii. ……

      iii. ……

      iv. ……

4. Have the action plans been implemented? ☐ Yes ☐No

   a. Please comment on the response: If yes Status of implementation; if not state reasons

      i. ………

         ………

      ii. ………

      iii. ………

5. What support, if any, did the Supervisor give in the implementation of the action plan?

   a. ……

   b. ……

   c. ……

   d. ………

6. Any comments from Trainee?

   a. ……

   b. ……

   c. ……

   d. ……

   e. ………

7. Best Practices Observed

   a. ……

   b. ………..

   c. ……

   d. ……

   e. ……

ACTION PLAN TEMPLATE: For developing an Action Plan for implementation to address identified gaps during follow-up visit

Name of Facility: ………………………………… Region: …………………………….. District: ……………………………………… Date of visit: ………………………………….

Name of Lead Supervisor: ………………………………………………………. Cadre: …………………….…. Contact Number: …………………………………………………….

Name of Facility In-charge/person met: …………………………………………….. Cadre: ……………………….… Contact Number: ………………………………………….

No.	Gaps/challenges identified	Action(s) taken by Supervisors	Action (s) to be taken by facility	Indicator (s)	Person Responsible	Person Accountable	When (specific date)
1							
2							
3							
4							
5							

Supplement to: Asiedu A, Haws R, Gyasi A, et al. Improving malaria case management and referral relationships at the primary care level in Ghana: evaluation of a quality assurance internship. *Glob Health Sci Pract*. 2023;11(6):e2300050. https://doi.org/10.9745/GHSP-D-23-00050

Supplement Table S1. HMIS Data Elements and Definitions

Data Element	Definition	Data Source
Disease (New Cases Only)	A count of the number of the individual conditions clients present with that are to be reported. New cases only, does not include cases on review.	Consulting room register
Uncomplicated Malaria Suspected	All Outpatient Department new cases that the that Clinician Suspects to be Malaria (including pregnant women)	Consulting room register
Uncomplicated Malaria Suspected Tested	Total number of suspected cases of malaria tested for malaria parasites (both rapid diagnostic tests and microscopy); including pregnant women	Consulting room register
Uncomplicated Malaria Tested Positive	Total number of suspected cases of malaria that tested positive for malaria parasites (both rapid diagnostic tests and microscopy); including pregnant women	Consulting room register
Uncomplicated Malaria not tested but Treated (Presumptive treatment)	Total number of suspected cases of malaria that were clinically diagnosed and treated without testing (including pregnant women)	Consulting room register
Uncomplicated Malaria tested negative but Treated (Non-adherence to negative test)	Total number of Suspected cases of malaria that tested negative for malaria parasites (both RDTs and Microscopy) but were treated as malaria	Consulting room register
IPT 1	Number of pregnant women given their first dose of SP at ANC	ANC register
IPT 2	Number of pregnant women given their second dose of SP at ANC	ANC register
IPT 3	Number of pregnant women given their third dose of SP at ANC	ANC register
IPT 4	Number of pregnant women given their fourth dose of SP at ANC	ANC register
IPT 5	Number of pregnant women given their fifth dose of SP at ANC	ANC register
ANC1	Number of pregnant women reporting for antenatal care for the first time to any health facility with their current pregnancy.	ANC register
Referral out	Number of malaria cases referred out	Consulting room register
Pre-referral treatment using rectal artesunate (Under-5)	Number of severe malaria cases under 5 provided prereferral treatment with rectal artesunate	Consulting room register
Confirmed cases	Number of confirmed/positive malaria cases	Consulting room register
Number of confirmed cases treated with ACTs	Number of positive malaria cases treated by ACT according to national guidelines	Consulting room register

Source: Monthly Outpatients Morbidity Returns in National HMIS SOP, 2020.

Supplement to: Asiedu A, Haws R, Gyasi A, et al. Improving malaria case management and referral relationships at the primary care level in Ghana: evaluation of a quality assurance internship. *Glob Health Sci Pract*. 2023;11(6):e2300050. https://doi.org/10.9745/GHSP-D-23-00050

Supplement Table S2. Malaria Indicators Using HMIS Data Elements

Indicator	Numerator data element	Denominator data element
Presumptive treatment	Uncomplicated Malaria not tested but Treated	Uncomplicated Malaria Suspected
Non-adherence to negative test results	Uncomplicated Malaria tested negative but Treated	Uncomplicated Malaria Suspected Tested
IPTp 3 Coverage	IPTp 3	ANC1
Testing rate for suspected uncomplicated malaria	Uncomplicated Malaria Suspected Tested	Uncomplicated Malaria Suspected
Percent of confirmed cases referred	Referral Out	Confirmed cases
Percent of Under-5 Cases Given Appropriate Pre-Referral Treatment	Pre-referral treatment using rectal artesunate (Under-5)	Referral out (Under-5 only)
Confirmed Cases Treated with ACTs Correctly	Number of confirmed malaria cases treated with ACTs	Confirmed cases
